# Causal analysis of the impact of serum 25-hydroxyvitamin D levels on laryngeal cancer: A two-sample mendelian randomization study

**DOI:** 10.1016/j.bjorl.2025.101705

**Published:** 2025-09-10

**Authors:** Bo Li, Cuiping She

**Affiliations:** a Central Hospital of Dalian University of Technology (Dalian Municipal Central Hospital), Department of Otorhinolaryngology Head and Neck Surgery, Dalian, China; b Dalian Medical University, Dalian, China; c Baotou Central Hospital, Baotou, China

**Keywords:** Serum 25-hydroxyvitamin D level, Laryngeal cancer, Genome-wide association study, Mendelian randomization, Single nucleotide polymorphism

## Abstract

**Objective:**

The levels of vitamin D in the human body are primarily measured through serum 25-hydroxyvitamin D (25(OH)D) levels. Observational studies suggest a potential association between the incidence of laryngeal cancer and vitamin D levels, but the causality remains unclear. This study aims to investigate the potential causal relationship between vitamin D levels and laryngeal cancer.

**Methods:**

This Mendelian Randomization (MR) study is based on large-scale GWAS (Genome-Wide Association Study) summary datasets. We selected two different datasets of 25(OH)D and conducted two two-sample univariable Mendelian Randomization (MR) analyses. Four different MR methods were applied, and a series of sensitivity analyses were performed. In addition, a two-sample Mendelian randomization was conducted to account for the confounding effect of smoking. Furthermore, we performed GO enrichment analyses on the SNPs used as instrumental variables.

**Results:**

The combined findings from both univariable MR analyses support a potential causal relationship between serum 25(OH)D levels and laryngeal cancer, suggesting that higher levels of vitamin D may have a protective effect against laryngeal cancer. Multivariable MR analysis showed that even after accounting for smoking as a confounding factor, the impact of 25(OH)D on laryngeal cancer remained significant. Enrichment analysis further indicated that 25(OH)D may inhibit the occurrence and progression of laryngeal cancer by regulating the metabolism of exogenous substances, lipid metabolism, and cellular responses to environmental stimuli.

**Conclusion:**

Higher serum 25-hydroxyvitamin D levels serve as a protective factor against laryngeal cancer, suggesting that increasing vitamin D levels may reduce the risk of laryngeal cancer.

**Level of evidence:**

This was a Mendelian randomized study with a level of evidence second only to clinical randomized trials, and higher than cohort and case-control studies.

## Highlights

• MR shows high serum 25(OH)D levels reduce laryngeal cancer risk.
• GWAS suggests vitamin D may have a protective role against laryngeal cancer.
• Vit D deficiency linked to higher laryngeal cancer risk, suggesting preventive.

## Introduction

Laryngeal Cancer (LC) is a common malignant tumor in the head and neck region, with an incidence rate of approximately 2.76 cases per 100,000 people and a mortality rate of 1.66 cases per 100,000 people worldwide. Over the past 30-years, both the incidence and prevalence rates have been increasing annually.^1^ The etiology of laryngeal cancer remains unclear and is currently believed to be the result of multiple carcinogenic factors working in synergy. In addition to well-known etiological factors such as smoking, excessive alcohol consumption, and HPV (Human Papillomavirus) infection, recent studies have also focused on vitamin D levels in the human body. However, the causal relationship between these factors and laryngeal cancer has not been fully established, necessitating further research to identify new preventive and therapeutic measures.

In recent years, the exponential increase in vitamin D testing has underscored the widespread and growing issue of vitamin D deficiency.^2^ Epidemiological studies have found that approximately 40% of Europeans are vitamin D deficient, with 13% being severely deficient.^3^ An increasing number of studies have demonstrated significant associations between vitamin D and cancer, particularly colorectal, prostate, and breast cancer. One study indicated that individuals with lower vitamin D levels have a 30%–50% increased risk of developing colorectal, prostate, and breast cancer.^4^ Recent molecular biology research has also revealed that the vitamin D system, in addition to maintaining calcium homeostasis, has roles in regulating immunity, reducing inflammation, and inhibiting cancer. The vitamin D system may inhibit cancer occurrence, growth, and metastasis through various molecular mechanisms, including regulating cell proliferation, differentiation, apoptosis, antioxidation, immune regulation, and cell communication and adhesion.

A study from Iran showed that vitamin D levels in laryngeal cancer patients were significantly lower than in healthy controls, suggesting that vitamin D may have a protective effect against laryngeal cancer.^5^ Another study found that vitamin D deficiency was common in advanced laryngeal cancer patients undergoing total laryngectomy and was associated with the development of pharyngocutaneous fistula.^6^ Conversely, a large-scale study in Finland found no association between serum 25-hydroxyvitamin D levels and the risk of head and neck cancer, highlighting the complexity of the relationship between vitamin D and laryngeal cancer.^7^ The findings on the relationship between vitamin D and laryngeal cancer are complex and inconsistent, necessitating further research.

Mendelian Randomization (MR) is an emerging method used to determine potential causal relationships between exposure factors and outcomes. Specific Single Nucleotide Polymorphisms (SNPs) are used as Instrumental Variables (IVs).^8^ Due to the random allocation of alleles during gamete formation, this design is less likely to be confounded or affected by reverse causality. With the advantages of this study design, MR can effectively reveal causal relationships between exposure and outcomes. Previously, MR designs have been used to explore the causal relationships between serum 25(OH)D and various diseases, such as major depression, diabetes, and multiple sclerosis,^9^, ^10^, ^11^ but have not been applied to study its effects on laryngeal cancer. Therefore, we conducted a two-sample MR study to investigate the causal relationship between serum 25(OH)D levels and laryngeal cancer.

**Fig. 1:**
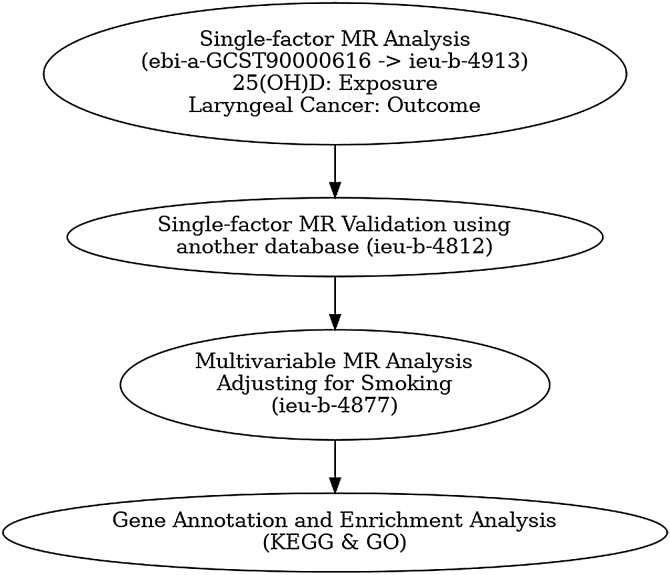
Flowchart of Mendelian Randomization (MR) analysis examining the causal relationship between 25(OH)D (vitamin D) and laryngeal cancer. The process starts with a single-factor MR analysis using the ebi-a-GCST90000616 dataset for 25(OH)D as the exposure and ieu-b-4913 for laryngeal cancer as the outcome. This is followed by validation using a different database (ieu-b-4812). A multivariable MR analysis is then performed to adjust for smoking (ieu-b-4877). Finally, gene annotation and enrichment analysis, including KEGG and GO, is conducted to interpret the genetic findings.

Our overall analysis process is as follows: First, we selected ebi-a-GCST90000616 as the primary dataset for the exposure factor 25(OH)D, and performed univariable Mendelian randomization analysis using the laryngeal cancer dataset ieu-b-4913, followed by sensitivity testing. We then used ieu-b-4812 as an auxiliary dataset for 25(OH)D to repeat the steps above for causal validation. Subsequently, we conducted multivariable MR analysis to account for potential confounding factors such as smoking, using the smoking dataset ieu-b-4877, and performed sensitivity analysis as well. Finally, we annotated the instrumental variables (SNPs) used in the MR analysis between the primary dataset and laryngeal cancer, and carried out GO enrichment analyses (Fig. 1: Flowchart).

## Methods

### Data sources

This study is based on large-scale GWAS summary datasets. All participants in the original studies provided informed consent, and this study only uses summary-level statistical data, thus requiring no additional ethical approval. In this study, all participants were of European descent. All statistical analyses were performed using R software (version 4.3.3) and were conducted through the TwosampleMR, MVMR, and BioMart packages.

In this research, we selected 25(OH)D as the exposure factor for Mendelian Randomization (MR) analysis, laryngeal cancer as the outcome, and smoking as a potential confounder. The exposure data were sourced from the IEU (MRC Integrative Epidemiology Unit) GWAS database, a globally leading repository of aggregated data on various health-related genetic factors. We chose two datasets related to 25(OH)D, using the dataset with ID ebi-a-GCST90000616 as the primary dataset and the dataset with ID ieu-b-4812 as an auxiliary and validation dataset. The outcome dataset for laryngeal cancer was sourced from the UK Biobank, with the specific ID ieu-b-4913. For the confounder, smoking-related data were obtained from the dataset with ID ieu-b-4877.

Detailed information and characteristics of all datasets are summarized in Table 1, and access to the databases can be obtained via the IEU GWAS resource platform (https://gwas.mrcieu.ac.uk).

**Table 1:** Summary of exposure, outcome, and potential confounders in mendelian randomization study.

Type	OPENGWAS_ID	Trait	Case	Control	Sample size
Exposure	ebi-a-GCST90000616	Serum 25-hydroxyvitamin D levels	NA	NA	417,580
Exposure	ieu-b-4812	25 hydroxyvitamin D level	NA	NA	441,291
Potential confounders	ieu-b-4877	Smoking initiation	NA	NA	607,291
Outcome	ieu-b-4913	Laryngeal cancer	273	372,016	372,289

### Extraction of genetic instrumental variables

MR analysis uses genetic variants as Instrumental Variables (IVs) to infer causal relationships between exposure and outcomes. To obtain unbiased estimates, the Single Nucleotide Polymorphisms (SNPs) chosen as exposure IVs must meet three key assumptions: 1) The IVs used in the analysis should be significantly associated with the exposure; 2) The IVs should be independent of confounding factors that might be related to both the exposure and the outcome; 3) The IVs should influence the outcome only through the exposure, and not through any other biological pathways (no horizontal pleiotropy).^12^ If all three assumptions are satisfied, the causal relationship between the exposure and the outcome can be calculated and is less likely to be influenced by unmeasured confounding factors.

#### Extraction of instrumental variables in univariable MR analysis

In the univariable MR analysis, we performed a series of steps to filter eligible SNPs. First, we extracted SNPs that were significantly associated with the exposure (p < 5 × 10^-8) and ensured that all instrument SNPs for the exposure were not in Linkage Disequilibrium (LD) (R^2 < 0.001 within 10,000 kb). Next, we harmonized the exposure and outcome SNPs to maintain consistency in the effect alleles. If a corresponding SNP was not available in the outcome data, a highly correlated SNP (LD proxy, LD > 0.8) was used as a substitute; if no proxy SNP was available, the SNP was excluded. To ensure consistency of the effect alleles between the exposure and outcome data, we set the allele frequency threshold to 0.3 for palindromic SNPs.

Subsequently, we conducted a global test using MR-PRESSO (Pleiotropy RESidual Sum and Outlier) to identify inconsistencies in the genetic associations of different genetic variants and removed outlier variants. Finally, we calculated the R^2 and F-statistics for each SNP and for the total set of SNPs. R^2 represents the proportion of variance in the exposure factor explained by the IV. The F-statistic was calculated to indicate the strength of the association between the instrument and the exposure of interest.^13^ An F-statistic of ≥ 10 suggests that the selected SNPs are valid and sufficiently strong.^13^ The R^2 and F-statistics were calculated using the following equations: $R^{2}=2\times (1-eaf)\times eaf\times \beta^{2}$; $F=(\frac{R^{2}}{1-R^{2}})(\frac{N-k-1}{k})$.

#### Extraction of instrumental variables in multivariable MR analysis

Simple two-sample univariable MR analysis is prone to horizontal pleiotropy. Therefore, we conducted multivariable MR analysis to eliminate potential confounding factors. The IV extraction process for multivariable MR analysis is as follows: 1) Extract the SNPs strongly associated with each exposure (p < 5e-8) and remove those in LD (R^2 < 0.001 within 10,000 kb). 2) Combine the SNPs from different exposures. 3) After merging the SNPs, remove duplicates and once again filter out those in LD (R^2 < 0.001 within 10,000 kb). 4) Harmonize the data for all exposure factors to ensure they are compared on the same effect allele, following the same harmonization process as in the univariable MR analysis. 5) Using the final set of SNPs, harmonize them again across multiple exposures and the outcome, compiling the corresponding beta and SE for both exposure and outcome. 6) Perform PRESSO tests and LASSO regression to exclude outliers and SNPs with multicollinearity. 7) Calculate the F-statistic for the total set of SNPs.

### Mendelian randomization analysis and results visualization

We primarily used the Inverse Variance Weighted (IVW) method for MR analysis to estimate causal effects.^14^ In addition, we applied weighted mode, weighted median, and MR-Egger regression methods as supplementary approaches to validate the causal relationship. These methods are considered robust and are commonly used to reliably analyze MR results.^15^^,^^16^ IVW computes the weighted average of the Wald ratios for individual SNPs and assumes that all instruments are valid, offering maximum statistical power but making it more susceptible to bias. In this study, the random effects model was applied in IVW, as it remains more conservative even when heterogeneity is detected. The MR-Egger regression model provides a relatively robust estimate independent of IV validity.^17^ However, MR-Egger is less precise and has lower statistical power. When at least half of the IVs are valid, the weighted median method can yield unbiased results. Thus, these methods serve as complementary approaches to the IVW results. Finally, we visualized the results using scatter plots, forest plots, and other graphical methods for clearer and more intuitive representation.

### Sensitivity analysis

In MR studies, sensitivity analysis is used to assess potential biases, including heterogeneity and horizontal pleiotropy. In this study, we used Cochran's *Q* test to detect heterogeneity and MR-Egger regression intercept to test for horizontal pleiotropy (where a significant intercept [p < 0.05] indicates horizontal pleiotropy).^18^^,^^19^ In addition, we used MR-PRESSO to exclude SNPs with pleiotropic outliers (p < 0.05).^17^^,^^20^ When outliers were detected, they were removed, and IVW estimation was re-performed to assess the robustness of the results. Lastly, we generated funnel plots to observe the symmetry of the scatter points and performed leave-one-out analysis to determine whether MR estimates were driven or biased by individual SNPs. For multivariable MR analysis, we also conducted LASSO regression to remove SNPs with multicollinearity.

### Gene annotation and enrichment analysis

In this study, we used BioMart for functional annotation to identify genes associated with 100 SNPs. Through BioMart, we obtained information on the gene functions, regulatory regions, and potential functional consequences of these rsIDs. Subsequently, using the clusterProfiler package, we converted Ensembl gene IDs into Entrez gene IDs and Gene Symbols, and performed GO enrichment analyses. These analyses helped us better understand the functional impact of these SNPs and their potential biological mechanisms.

## Result

### Univariable mendelian randomization analysis of 25(OH)D primary dataset and laryngeal cancer

#### Analysis results

**Fig. 2:**
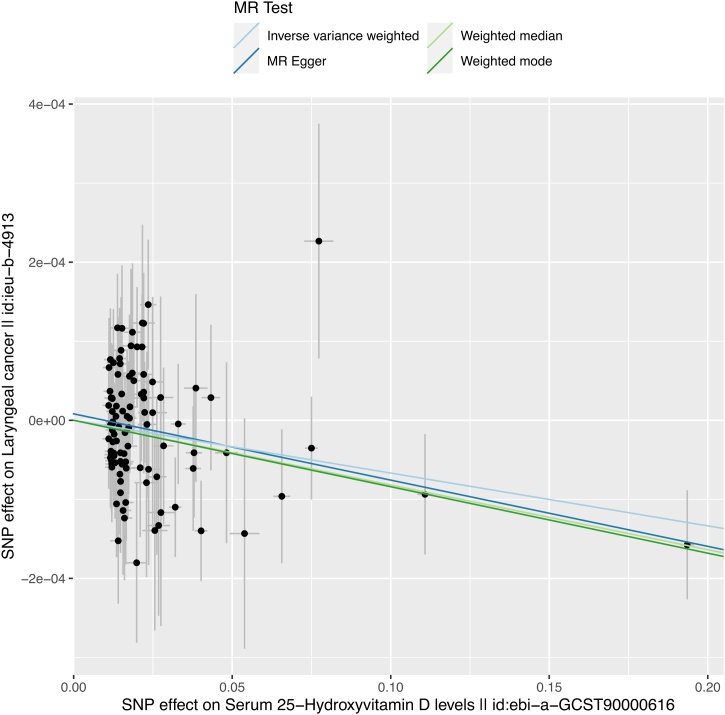
The scatter plot illustrates the SNP effect between serum 25-hydroxyvitamin D levels and laryngeal cancer from the primary dataset (ebi-a-GCST90000616, ieu-b-4913). Results from the four Mendelian Randomization (MR) methods (Inverse Variance Weighted, MR Egger, Weighted Median, and Weighted Mode) show that most SNP effect sizes cluster around the linear relationship, suggesting a potential causal association between vitamin D and laryngeal cancer, with the Inverse Variance Weighted (IVW) method showing particularly significant results.

After filtering, 100 independent SNPs were selected as genetic instrumental variables. All Instrumental Variables (IVs) were strongly associated with the exposure factor (all SNPs had p-values less than 5e-8, with the minimum F-statistic for a single SNP being 29.77976, and the overall F-statistic being 184). Detailed information on the 100 SNPs can be found in Supplementary Table 1 and Supplementary Table 2. The MR analysis results showed a causal relationship between serum 25-hydroxyvitamin D levels and laryngeal cancer (IVW: p < 0.01). The other three methods also supported this conclusion (p < 0.05). The specific results are shown in Table 2. A linear regression analysis was conducted, and a scatter plot was drawn, indicating that as serum 25-hydroxyvitamin D levels increase, the risk of laryngeal cancer decreases (Fig. 2). After unit conversion, the analysis revealed that for every 20 ng/mL increase in vitamin D concentration, the Odds Ratio (OR) for laryngeal cancer risk was approximately 0.967, with a 95% Confidence Interval of [0.945, 0.990] (OR = 0.967, 95% CI [0.945, 0.990], p < 0.01). However, due to the small sample size of the case group in the outcome variable and the inability of the existing laryngeal cancer database to provide a larger sample size, the effect in the analysis was diluted, resulting in a smaller OR value. This also limited the statistical power to some extent, so it is hoped that future studies with larger databases can confirm the accuracy of these findings.

**Table 2:** Evaluation results of causal relationship between exposure factor and outcome variable using different methods.

Method	nsnp	β	se	p-val
Inverse variance weighted	100	−6.677e-4	2.352e-4	0.004526
Weighted mode	100	−8.405e-4	2.909e-4	0.004741
MR Egger	100	−8.38e-4	3.183e-4	0.009832
Weighted median	100	−8.186e-4	3.472e-4	0.0184

Notes:

1. The beta coefficients for all methods are negative, indicating that an increase in the exposure factor may be associated with a reduced risk of the outcome variable.

2. The p-values for all methods are less than 0.05, indicating that the results are statistically significant.

3. The results show good consistency among the estimates from different methods.

#### Sensitivity analysis and results visualization

Cochran's *Q* statistic was used to test for heterogeneity, with p > 0.05 indicating no significant heterogeneity (i.e., the causal estimates of all SNPs are consistent). In this study, results showed low heterogeneity (Supplementary Table 3). When using Egger regression to analyze the data, the p-value tested whether the Egger regression intercept was significantly different from zero. If p < 0.05, it indicates the presence of horizontal pleiotropy, meaning that some genetic variants affect the outcome through pathways other than the exposure. In this study, the Egger regression intercept p-value was > 0.05, indicating no significant horizontal pleiotropy (Supplementary Table 4). No significant outliers were detected in the MR-PRESSO analysis.

We visualized the results and created plots. The forest plot (Supplementary Fig. 1) shows the effect estimates of each SNP and their uncertainties, with regression lines representing the causal effect estimates from different methods. Both the IVW and MR-Egger methods show negative effect estimates, supporting the causal association between an increase in the exposure factor and a decrease in the risk of the outcome variable. The MR-Egger method results show no significant directional pleiotropy, enhancing the reliability of the causal inference. The funnel plot (Supplementary Fig. 2) tested the horizontal pleiotropy of the MR analysis by examining the symmetry of the scatter points. The results showed symmetry on both sides, indicating reliable results without the influence of horizontal pleiotropy. Additionally, a leave-one-out analysis (Supplementary Fig. 3) showed that no single SNP significantly influenced the overall results. Overall, the results of this study suggest that there may be a significant causal relationship between serum 25-hydroxyvitamin D levels and the risk of laryngeal cancer.

### Univariable MR analysis validation using a different exposure dataset

#### Results

To validate the consistency of the results, we selected another 25(OH)D dataset from a different data source as the exposure factor and performed a univariable MR analysis with the outcome dataset. The dataset ID is ieu-b-4812. In this analysis, we selected 94 SNPs as Instrumental Variables (IVs). Compared to the 100 SNPs selected previously, only a few were overlapping. All IVs were strongly associated with the exposure factor (all SNPs had p-values less than 5e-8, with the minimum F-statistic for a single SNP being 29.75448, and the overall F-statistic being 143). Detailed information on the 94 SNPs can be found in Supplementary Tables 5 and 6.

**Fig. 3:**
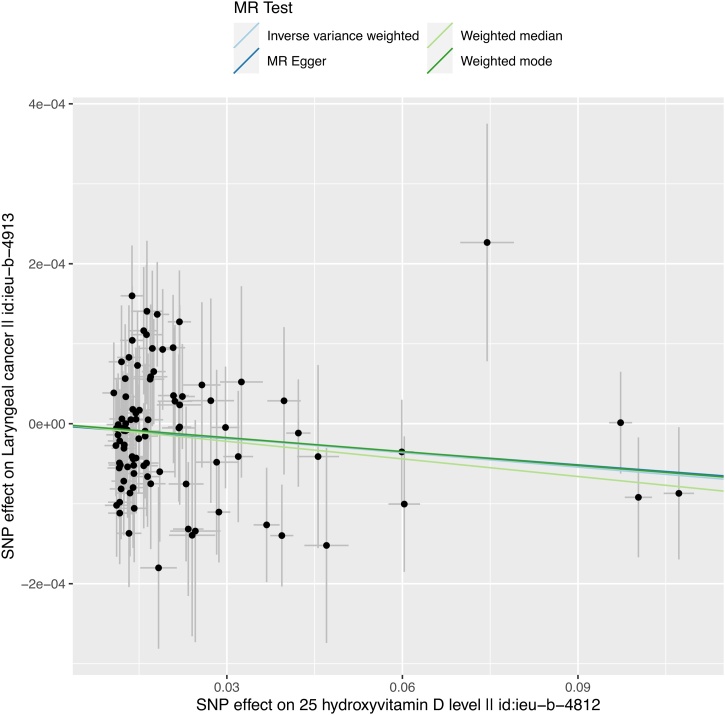
The scatter plot illustrates the SNP effect between 25-hydroxyvitamin D levels and laryngeal cancer from the validation dataset (ieu-b-4812, ieu-b-4913). Results from the four MR methods show that the distribution of SNP effect sizes in the validation dataset is similar to that in the primary dataset, further validating the potential causal relationship between vitamin D levels and laryngeal cancer. The consistency of the Inverse Variance Weighted method's results in both datasets supports the findings from the primary analysis.

The IVW analysis indicated a causal relationship between serum 25-hydroxyvitamin D levels and laryngeal cancer (IVW: β = −6.028e-4; SE = 2.773e-4; p < 0.05). The direction of the estimates was consistent across the other three methods. The specific results are shown in Table 3. We conducted a linear regression analysis and plotted a scatter plot, showing that as serum 25-hydroxyvitamin D levels increased, the risk of laryngeal cancer decreased (Fig. 3). After unit conversion, the analysis results indicated that for every 20 ng/mL increase in vitamin D concentration, the Odds Ratio (OR) for laryngeal cancer risk was approximately 0.9703, with a 95% Confidence Interval of [0.9443, 0.9970] (OR = 0.9703, 95% CI [0.9443, 0.9970], p < 0.05). However, due to the small sample size of the case group in the outcome variable, so it is hoped that future studies with larger databases can confirm the accuracy of these findings.

**Table 3:** Results of different methods evaluating the causal relationship between the exposure factor and the outcome variable in the validation dataset.

Methods	nsnp	β	SE	p-val
IVW	94	−6.028e-4	2.773e-4	0.02972
Weighted mode	94	−5.797e-4	3.596e-4	0.1103
MR Egger	94	−5.508e-4	4.262e-4	0.1994
Weighted median	94	−7.331e-4	4.341e-4	0.09124

Notes:

1. The β coefficients for all methods are negative, indicating that an increase in the exposure factor may be associated with a decreased risk of the outcome variable.

2. The p-value for the IVW method is less than 0.05, and the direction of the estimates is consistent across other methods.

#### Sensitivity analysis and results visualization

We used Cochran's *Q* statistic to test for heterogeneity. The results of this study showed low heterogeneity (see Supplementary Table 7). Egger regression was applied for analysis. In this study, the p-value for the Egger regression intercept was greater than 0.05, indicating no significant horizontal pleiotropy (see Supplementary Table 8). No significant outliers were detected in the MR-PRESSO analysis.

We visualized the results and created plots. The forest plot (Supplementary Fig. 4), funnel plot (Supplementary Fig. 5), and leave-one-out analysis (Supplementary Fig. 6) were largely consistent with the previous findings.

### Multivariable MR analysis to eliminate confounding factors

To rule out the potential confounder ‒ smoking, we conducted a Multivariable MR (MVMR) analysis. The results of the analysis are shown in Table 4. The primary IVW method of the multivariable MR analysis showed that even after accounting for the confounding factor of smoking, the protective effect of 25(OH)D on laryngeal cancer remained significant (p < 0.05). It is worth noting that, due to the small sample size in this analysis, the effect was diluted, resulting in a smaller OR value.

**Table 4:** Results of the analysis.

Adjustment	nSNP	Methods	p
Smoking intiation	84	Median	0.01
	84	Lasso	0.02
	84	IVW	0.02
	84	Egger	0.03

In this MVMR analysis, 84 SNPs were used, and no outliers or SNPs with multicollinearity were detected through PRESSO testing and LASSO regression. The total F-statistic is presented in Supplementary Table 9. Cochran's *Q* test detected no heterogeneity (p > 0.05).

### Functional annotation

**Fig. 4:**
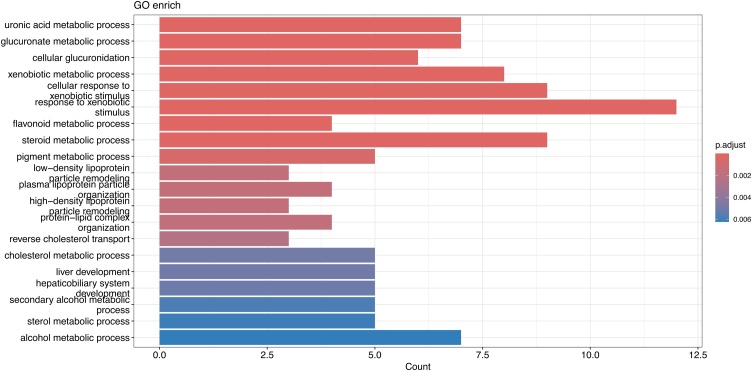
The bar plot shows the biological processes obtained through SNP gene annotation and GO enrichment analysis. Each bar represents the extent of gene enrichment in different biological processes. The x-axis indicates the number of genes involved in these processes, and the color gradient reflects statistical significance.

To explore the potential mechanisms by which 25(OH)D influences laryngeal cancer, we conducted GO enrichment analyses. GO enrichment analysis provided insights into the functions of the related genes from three perspectives: molecular function, cellular components, and biological processes. KEGG enrichment analysis enabled us to better understand the signaling pathways of these genes and their specific roles in the occurrence and development of laryngeal cancer. Our results suggest that 25(OH)D may inhibit the occurrence and progression of laryngeal cancer by regulating the metabolism of exogenous substances, lipid metabolism, and cellular responses to environmental stimuli (Supplementary Tables 10–13) (Fig. 4). These metabolic and regulatory pathways, which are abnormally active in cancer, may accelerate cancer cell proliferation, uncontrolled growth, and contribute to drug resistance, ultimately driving the progression of laryngeal cancer.

## Discussion

This is the first study to explore the causal relationship between 25(OH)D and Laryngeal Cancer (LC) using a two-sample MR approach based on large-scale GWAS data. Our study suggests a potential causal relationship between serum 25(OH)D levels and laryngeal cancer, which requires further research for validation.

Laryngeal Cancer (LC), being one of the common malignant tumors in the head and neck region, is of significant importance for in-depth exploration. The pathological type of laryngeal cancer is mainly squamous cell carcinoma, accounting for more than 90%. According to the Global Health Data Exchange database, laryngeal cancer causes approximately 3.28 million Disability-Adjusted Life Years (DALYs) annually, with its incidence and prevalence increasing by 12% and 24%, respectively, over the past 30-years.^1^ In several studies conducted on different populations, vitamin D deficiency has been considered a potential cause of laryngeal cancer. In a Danish prospective cohort study by Afzal et al., which included 122 HNC (head and neck cancer) patients, a 50% reduction in 25(OH)D levels was associated with an HR (Hazard Ratio) of 1.20 for any tobacco-related cancer.^21^ In the EPIC (European Prospective Investigation into Cancer and Nutrition) cohort study, a case-control study involving 350 HNC patients and their individually matched controls, Fanidi et al.^22^ found that doubling the plasma levels of 25(OH)D significantly reduced the cancer risk in HNC patients compared to the control group. Conversely, a small Danish study investigating 38 cases of Head and Neck Squamous Cell Carcinoma (HNSCC) found no significant association between vitamin D levels and HNSCC incidence.^23^ Another larger Finnish study of 348 new HNSCC cases also found no association between serum vitamin D levels and HNSCC risk.^7^

These inconsistent conclusions may stem from various confounding factors, such as different study designs and populations. In our study, we used Mendelian randomization analysis. This method allows the use of genetic instruments to infer causal relationships in a potentially causal manner, avoiding bias due to confounding and estimating the assumed causal relationship under different conditions. To control for the effects of horizontal pleiotropy and heterogeneity on the MR analysis results, we also conducted a series of sensitivity analyses. We also conducted a multivariable Mendelian randomization analysis to account for the potential confounding effect of smoking. Our study suggests a potential causal relationship between serum 25(OH)D levels and laryngeal cancer, which requires further research for validation.

To explore the potential mechanisms by which 25(OH)D influences laryngeal cancer, we performed GO enrichment analyses on the instrumental variable SNPs used in the primary dataset. We found that 25(OH)D may inhibit the occurrence and development of laryngeal cancer by regulating the metabolism of exogenous substances, lipid metabolism, and cellular responses to environmental stimuli. These metabolic and regulatory pathways, which are abnormally active in cancer, may accelerate cancer cell proliferation, uncontrolled growth, and lead to drug resistance, ultimately driving the progression of laryngeal cancer.

To date, the mechanisms linking vitamin D to laryngeal cancer remain inconclusive. As a steroid hormone, vitamin D may play an important role in head and neck squamous cell carcinoma through the following pathways. Firstly, in vitro cell experiments have found that treating squamous cell carcinoma cell lines with different concentrations of vitamin D can reduce cell proliferation.^24^ Vitamin D also upregulates several cell cycle checkpoint inhibitors, such as p21, p18, and p27.^25^, ^26^, ^27^ Telomerase upregulation is common in HNSCC, and vitamin D treatment can inhibit the expression of Telomerase Reverse Transcriptase (TERT) in vitro.^27^ Additionally, in HNSCC in vitro models, vitamin D promotes cell differentiation, enhances the upregulation of DNA damage response pathways, and inhibits invasive and metastatic activities.^28^, ^29^, ^30^

Secondly, immune regulation: HNSCC is often accompanied by tumor-driven immune dysfunction, creating an inflammatory environment that favors malignancy.^31^ Studies have shown that the immune response to HNSCC is sensitive to vitamin D levels.^32^^,^^33^ Vitamin D treatment can reduce the presence of immunosuppressive immature dendritic cells in tumors while increasing the infiltration of mature dendritic cells.^34^ Higher levels of vitamin D in HNSCC patients are associated with increased infiltration of CD4+ T-cells in the tumor and surrounding stroma, correlating with longer overall survival.^35^

For decades, the anticancer effects of vitamin D have been studied in various malignancies, yielding mixed results, thus the anticancer effects of vitamin D remain unclear.^36^ Our study suggests a potential causal relationship between serum 25(OH)D levels and laryngeal cancer. In this study, both the GWAS data for laryngeal cancer and serum 25(OH)D genes were derived from European populations, avoiding the effects of population stratification. In this process, we selected two different datasets for 25(OH)D and performed two separate two-sample univariable Mendelian randomization analyses, applying four different MR methods and conducting a series of sensitivity analyses. Additionally, we performed two-sample Mendelian randomization to account for the confounding effect of smoking. The results of the comprehensive analysis consistently suggest a potential causal relationship between serum 25(OH)D levels and laryngeal cancer. Vitamin D supplementation may reduce the risk of laryngeal cancer, and our study highlights the potential benefit of vitamin D supplementation as a preventive measure for laryngeal cancer.

Our study also has certain limitations: firstly, due to the small sample size of the case group in the outcome variable and the inability of the existing laryngeal cancer database to provide a larger sample size, the effect in the analysis was diluted, resulting in a smaller OR value. This also limited the statistical power to some extent. Secondly, we established the presumed causal relationship between 25(OH)D and LC in individuals of European ancestry. Future studies need to extend our conclusions to other populations. Thirdly, the mechanisms by which increased serum 25(OH)D levels reduce the risk of laryngeal cancer require further validation. Fourthly, we used only summary-level statistical data, which does not allow for stratified analysis.

## Conclusion

This study is the first to employ a two-sample Mendelian Randomization (MR) approach using large-scale GWAS data to explore the causal relationship between serum 25-hydroxyvitamin D (25(OH)D) levels and laryngeal cancer risk. The research results suggest a potential causal relationship between serum 25(OH)D levels and laryngeal cancer, indicating that higher levels of vitamin D may have a protective effect against laryngeal cancer. Given the rising incidence of laryngeal cancer and the widespread prevalence of vitamin D deficiency, this study highlights the potential benefit of vitamin D supplementation as a preventive measure for laryngeal cancer.

Future studies should incorporate larger sample-sized databases for analysis, which will help to draw more reliable conclusions and further clarify the relationship between 25(OH)D and laryngeal cancer. Additionally, stratified analysis based on individual data may provide deeper insights into the relationship between 25(OH)D and laryngeal cancer in different populations.

## ORCID ID

Bo Li: 0009-0005-4769-4630

Cuiping She: 0000-0002-5431-8925

## CRediT authorship contribution statement

Bo Li and Cuiping She designed the study. Bo Li conducted the research, analyzed the data, and wrote the manuscript. Cuiping She reviewed the intellectual content of the manuscript. All authors contributed to the article and approved the version submitted.

## Funding

This manuscript did not receive any funding.

## Data availability

The original contributions presented in the study are included in the article/supplementary material. Further inquiries can be directed to the corresponding author.

## Declaration of competing interest

This research did not receive any specific grant from funding agencies in the public, commercial, or not-for-profit sectors. No potential conflict of interest was reported by the authors.

## References

[1] Nocini R., Molteni G., Mattiuzzi C., Lippi G. (2020). Updates on larynx cancer epidemiology. Chin J Cancer Res.

[2] Crowe F.L., Jolly K., MacArthur C. (2019). Trends in the incidence of testing for vitamin D deficiency in primary care in the UK: a retrospective analysis of The Health Improvement Network (THIN), 2005-2015. BMJ Open.

[3] Amrein K., Scherkl M., Hoffmann M. (2020). Vitamin D deficiency 2.0: an update on the current status worldwide. Eur J Clin Nutr.

[4] Holick M.F. (2007). Vitamin D deficiency. N Engl J Med.

[5] Khadivi E., Moghaddas N., Rasti Boroujeni H. (2023). The relationship between vitamin D deficiency and laryngeal cancer: a case-control study in Mashhad, Iran. Research Article. Int J Cancer Manag.

[6] Iravani K., Khosravi Y., Doostkam A., Soltaniesmaeili A. (2024). Vitamin D deficiency in advanced laryngeal cancer and its association with pharyngocutaneous fistula following total laryngectomy. Curr Drug Saf.

[7] Arem H., Weinstein S.J., Horst R.L. (2011). Serum 25-hydroxyvitamin D and risk of oropharynx and larynx cancers in Finnish men. Cancer Epidemiol Biomarkers Prev.

[8] Emdin C.A., Khera A.V., Kathiresan S. (2017). Mendelian randomization. JAMA.

[9] Michaëlsson K., Melhus H., Larsson S.C. (2018). Serum 25-hydroxyvitamin D concentrations and major depression: a mendelian randomization study. Nutrients.

[10] Afzal S., Brøndum-Jacobsen P., Bojesen S.E., Nordestgaard B.G. (2014). Vitamin D concentration, obesity, and risk of diabetes: a mendelian randomisation study. Lancet Diabetes Endocrinol.

[11] Wang R. (2022). Mendelian randomization study updates the effect of 25-hydroxyvitamin D levels on the risk of multiple sclerosis. J Transl Med.

[12] Davies N.M., Holmes M.V., Davey Smith G. (2018). Reading Mendelian randomisation studies: a guide, glossary, and checklist for clinicians. BMJ.

[13] Burgess S., Thompson S.G. (2011). Avoiding bias from weak instruments in Mendelian randomization studies. Int J Epidemiol.

[14] Burgess S., Butterworth A., Thompson S.G. (2013). Mendelian randomization analysis with multiple genetic variants using summarized data. Genet Epidemiol.

[15] Chen X., Kong J., Pan J. (2021). Kidney damage causally affects the brain cortical structure: a mendelian randomization study. EBioMedicine.

[16] Chen X., Hong X., Gao W. (2022). Causal relationship between physical activity, leisure sedentary behaviors and COVID-19 risk: a mendelian randomization study. J Transl Med.

[17] Ong J.S., MacGregor S. (2019). Implementing MR-PRESSO and GCTA-GSMR for pleiotropy assessment in Mendelian randomization studies from a practitioner’s perspective. Genet Epidemiol.

[18] Bowden J., Davey Smith G., Burgess S. (2015). Mendelian randomization with invalid instruments: effect estimation and bias detection through Egger regression. Int J Epidemiol.

[19] Burgess S., Thompson S.G. (2017). Interpreting findings from Mendelian randomization using the MR-Egger method. Eur J Epidemiol.

[20] Bowden J., Spiller W., Del Greco M.F. (2018). Improving the visualization, interpretation and analysis of two-sample summary data Mendelian randomization via the Radial plot and Radial regression. Int J Epidemiol.

[21] Afzal S., Bojesen S.E., Nordestgaard B.G. (2013). Low plasma 25-hydroxyvitamin D and risk of tobacco-related cancer. Clin Chem.

[22] Fanidi A., Muller D.C., Midttun Ø. (2016). Circulating vitamin D in relation to cancer incidence and survival of the head and neck and oesophagus in the EPIC cohort. Sci Rep.

[23] Skaaby T., Husemoen L.L., Thuesen B.H. (2014). Prospective population-based study of the association between serum 25-hydroxyvitamin-D levels and the incidence of specific types of cancer. Cancer Epidemiol Biomarkers Prev.

[24] Enepekides D.J., Black M.J., White J.H. (1999). The independent and combined effects of RAR-, RXR-, and VDR-selective ligands on the growth of squamous cell carcinoma in vitro. J Otolaryngol.

[25] Hager G., Kornfehl J., Knerer B., Weigel G., Formanek M. (2004). Molecular analysis of p21 promoter activity isolated from squamous carcinoma cell lines of the head and neck under the influence of 1,25(OH)2 vitamin D3 and its analogs. Acta Otolaryngol.

[26] Hager G., Formanek M., Gedlicka C., Thurnher D., Knerer B., Kornfehl J. (2001). 1,25(OH)2 vitamin D3 induces elevated expression of the cell cycle-regulating genes P21 and P27 in squamous carcinoma cell lines of the head and neck. Acta Otolaryngol.

[27] Gedlicka C., Hager G., Weissenböck M. (2006). 1,25(OH)2Vitamin D3 induces elevated expression of the cell cycle inhibitor p18 in a squamous cell carcinoma cell line of the head and neck. J Oral Pathol Med.

[28] Chiang K.C., Yeh C.N., Hsu J.T. (2013). MART-10, a novel vitamin D analog, inhibits head and neck squamous carcinoma cells growth through cell cycle arrest at G0/G1 with upregulation of p21 and p27 and downregulation of telomerase. J Steroid Biochem Mol Biol.

[29] Yang S.W., Tsai C.Y., Pan Y.C. (2016). MART-10, a newly synthesized vitamin D analog, represses metastatic potential of head and neck squamous carcinoma cells. Drug Des Devel Ther.

[30] Lin R., Nagai Y., Sladek R. (2002). Expression profiling in squamous carcinoma cells reveals pleiotropic effects of vitamin D3 analog EB1089 signaling on cell proliferation, differentiation, and immune system regulation. Mol Endocrinol.

[31] Nasser H., St John M. (2018). Immunotherapeutic approaches to head and neck cancer. Crit Rev Oncog.

[32] Almand B., Clark J.I., Nikitina E. (2001). Increased production of immature myeloid cells in cancer patients: a mechanism of immunosuppression in cancer. J Immunol.

[33] Walker D.D., Reeves T.D., de Costa A.M., Schuyler C., Young M.R. (2012). Immunological modulation by 1α,25-dihydroxyvitamin D3 in patients with squamous cell carcinoma of the head and neck. Cytokine.

[34] Badoual C., Hans S., Rodriguez J. (2006). Prognostic value of tumor-infiltrating CD4+ T-cell subpopulations in head and neck cancers. Clin Cancer Res.

[35] Bochen F., Balensiefer B., Körner S. (2018). Vitamin D deficiency in head and neck cancer patients - prevalence, prognostic value and impact on immune function. Oncoimmunology.

[36] Garland C.F., Garland F.C. (2006). Do sunlight and vitamin D reduce the likelihood of colon cancer?. Int J Epidemiol.

