# Supplementary material for: Causal analysis of the impact of serum 25-hydroxyvitamin D levels on laryngeal cancer: A two-sample mendelian randomization study
Source: Braz J Otorhinolaryngol. 2025 Sep 10;92(1):101705. doi: 10.1016/j.bjorl.2025.101705 (PMC12827714; doi:10.1016/j.bjorl.2025.101705)

**BJORL-D-24-00231_ Supplementary Material**

**Supplementary Table 1** Instrumental variables.

| **Exposure** | **SNP** | **effect_allele** | **other_allele** | **beta** | **se** | **pval** |
| --- | --- | --- | --- | --- | --- | --- |
| Serum 25-Hydroxyvitamin D levels | rs12123821 | T | C | 0.077 | 0.005 | 2.87e-63 |
| Serum 25-Hydroxyvitamin D levels | rs10908469 | C | A | 0.016 | 0.002 | 2.41e-13 |
| Serum 25-Hydroxyvitamin D levels | rs6671730 | A | G | -0.015 | 0.002 | 7.76e-14 |
| Serum 25-Hydroxyvitamin D levels | rs6672758 | T | C | 0.016 | 0.002 | 2.98e-11 |
| Serum 25-Hydroxyvitamin D levels | rs182050989 | T | C | -0.034 | 0.006 | 1.68e-08 |
| Serum 25-Hydroxyvitamin D levels | rs61816766 | C | T | 0.087 | 0.006 | 3.05e-49 |
| Serum 25-Hydroxyvitamin D levels | rs55707527 | T | G | 0.015 | 0.002 | 5.27e-10 |
| Serum 25-Hydroxyvitamin D levels | rs2131925 | T | G | -0.022 | 0.002 | 5.84e-27 |
| Serum 25-Hydroxyvitamin D levels | rs7528419 | G | A | 0.020 | 0.002 | 1.75e-17 |
| Serum 25-Hydroxyvitamin D levels | rs2642439 | G | A | -0.015 | 0.002 | 1.47e-12 |
| Serum 25-Hydroxyvitamin D levels | rs512083 | C | T | 0.012 | 0.002 | 2.64e-09 |
| Serum 25-Hydroxyvitamin D levels | rs35408430 | T | C | -0.022 | 0.002 | 1.72e-25 |
| Serum 25-Hydroxyvitamin D levels | rs6724965 | G | A | -0.017 | 0.003 | 8.76e-11 |
| Serum 25-Hydroxyvitamin D levels | rs2012736 | A | C | -0.048 | 0.004 | 1.14e-40 |
| Serum 25-Hydroxyvitamin D levels | rs1260326 | C | T | 0.022 | 0.002 | 3.46e-28 |
| Serum 25-Hydroxyvitamin D levels | rs7569755 | A | G | 0.013 | 0.002 | 6.15e-10 |
| Serum 25-Hydroxyvitamin D levels | rs13011615 | T | A | 0.017 | 0.003 | 8.8e-09 |
| Serum 25-Hydroxyvitamin D levels | rs1047891 | A | C | -0.014 | 0.002 | 1.38e-10 |
| Serum 25-Hydroxyvitamin D levels | rs6547409 | T | C | 0.028 | 0.005 | 1.19e-09 |
| Serum 25-Hydroxyvitamin D levels | rs727857 | A | G | -0.011 | 0.002 | 1.66e-08 |
| Serum 25-Hydroxyvitamin D levels | rs58387006 | C | A | -0.014 | 0.002 | 1.09e-08 |
| Serum 25-Hydroxyvitamin D levels | rs6438900 | G | C | 0.013 | 0.002 | 3.53e-09 |
| Serum 25-Hydroxyvitamin D levels | rs9861009 | C | T | 0.015 | 0.002 | 3.57e-11 |
| Serum 25-Hydroxyvitamin D levels | rs6782190 | A | G | -0.019 | 0.002 | 2.32e-20 |
| Serum 25-Hydroxyvitamin D levels | rs2246832 | T | A | 0.016 | 0.002 | 2.85e-16 |
| Serum 25-Hydroxyvitamin D levels | rs71297391 | T | C | 0.025 | 0.005 | 4.84e-08 |
| Serum 25-Hydroxyvitamin D levels | rs6834488 | T | C | -0.014 | 0.002 | 3.26e-13 |
| Serum 25-Hydroxyvitamin D levels | rs4364259 | A | G | 0.016 | 0.002 | 2.88e-11 |
| Serum 25-Hydroxyvitamin D levels | rs4616820 | T | C | -0.012 | 0.002 | 5.3e-10 |
| Serum 25-Hydroxyvitamin D levels | rs3890624 | G | A | 0.012 | 0.002 | 1.32e-08 |
| Serum 25-Hydroxyvitamin D levels | rs111978466 | T | C | 0.072 | 0.008 | 4.48e-21 |
| Serum 25-Hydroxyvitamin D levels | rs1352846 | G | A | -0.194 | 0.002 | 1e-200 |
| Serum 25-Hydroxyvitamin D levels | rs62318873 | T | C | 0.054 | 0.005 | 1.93e-30 |
| Serum 25-Hydroxyvitamin D levels | rs11249525 | T | C | -0.032 | 0.002 | 1.1e-59 |
| Serum 25-Hydroxyvitamin D levels | rs293435 | T | C | -0.018 | 0.002 | 5.1e-16 |
| Serum 25-Hydroxyvitamin D levels | rs189407772 | G | A | 0.053 | 0.007 | 2.03e-15 |
| Serum 25-Hydroxyvitamin D levels | rs75765985 | A | G | 0.053 | 0.008 | 9.22e-12 |
| Serum 25-Hydroxyvitamin D levels | rs78649910 | A | T | -0.020 | 0.003 | 5.61e-10 |
| Serum 25-Hydroxyvitamin D levels | rs1966478 | C | T | -0.012 | 0.002 | 1.09e-08 |
| Serum 25-Hydroxyvitamin D levels | rs72834856 | G | T | -0.024 | 0.004 | 4.66e-10 |
| Serum 25-Hydroxyvitamin D levels | rs28407950 | T | C | -0.014 | 0.002 | 9.41e-10 |
| Serum 25-Hydroxyvitamin D levels | rs9490317 | C | T | 0.011 | 0.002 | 2.46e-08 |
| Serum 25-Hydroxyvitamin D levels | rs143069752 | A | T | 0.022 | 0.004 | 3.39e-08 |
| Serum 25-Hydroxyvitamin D levels | rs2248551 | A | G | -0.021 | 0.003 | 6.67e-16 |
| Serum 25-Hydroxyvitamin D levels | rs9476310 | T | C | 0.012 | 0.002 | 3.45e-09 |
| Serum 25-Hydroxyvitamin D levels | rs7784802 | T | A | 0.014 | 0.002 | 1.23e-11 |
| Serum 25-Hydroxyvitamin D levels | rs2346264 | C | A | -0.015 | 0.002 | 1.17e-09 |
| Serum 25-Hydroxyvitamin D levels | rs41301394 | T | C | 0.012 | 0.002 | 3.19e-08 |
| Serum 25-Hydroxyvitamin D levels | rs1858889 | C | A | 0.011 | 0.002 | 1.28e-08 |
| Serum 25-Hydroxyvitamin D levels | rs10085881 | C | T | -0.015 | 0.002 | 2.17e-11 |
| Serum 25-Hydroxyvitamin D levels | rs12056768 | G | T | -0.022 | 0.002 | 2.76e-29 |
| Serum 25-Hydroxyvitamin D levels | rs57459725 | G | C | -0.017 | 0.003 | 3.43e-09 |
| Serum 25-Hydroxyvitamin D levels | rs804281 | G | A | 0.015 | 0.002 | 1.75e-14 |
| Serum 25-Hydroxyvitamin D levels | rs34726834 | T | C | 0.015 | 0.002 | 8.24e-11 |
| Serum 25-Hydroxyvitamin D levels | rs700065 | T | G | -0.018 | 0.003 | 6.6e-11 |
| Serum 25-Hydroxyvitamin D levels | rs532436 | A | G | -0.018 | 0.003 | 2.1e-12 |
| Serum 25-Hydroxyvitamin D levels | rs13284054 | C | T | 0.018 | 0.003 | 4.6e-09 |
| Serum 25-Hydroxyvitamin D levels | rs10822145 | T | C | -0.012 | 0.002 | 5.71e-10 |
| Serum 25-Hydroxyvitamin D levels | rs34993776 | T | C | 0.016 | 0.002 | 6.99e-11 |
| Serum 25-Hydroxyvitamin D levels | rs4418728 | T | G | 0.012 | 0.002 | 1.05e-09 |
| Serum 25-Hydroxyvitamin D levels | rs575976 | G | A | 0.016 | 0.003 | 1.06e-09 |
| Serum 25-Hydroxyvitamin D levels | rs33981819 | G | T | 0.012 | 0.002 | 4.09e-09 |
| Serum 25-Hydroxyvitamin D levels | rs2847500 | A | G | -0.022 | 0.003 | 1.15e-13 |
| Serum 25-Hydroxyvitamin D levels | rs10766281 | G | A | 0.015 | 0.002 | 7.46e-12 |
| Serum 25-Hydroxyvitamin D levels | rs964184 | C | G | 0.043 | 0.003 | 2.03e-50 |
| Serum 25-Hydroxyvitamin D levels | rs36037728 | T | C | -0.058 | 0.006 | 2.5e-21 |
| Serum 25-Hydroxyvitamin D levels | rs77037130 | A | G | -0.067 | 0.008 | 4.06e-17 |
| Serum 25-Hydroxyvitamin D levels | rs116970203 | A | G | -0.377 | 0.006 | 1e-200 |
| Serum 25-Hydroxyvitamin D levels | rs7128011 | A | G | -0.075 | 0.002 | 1e-200 |
| Serum 25-Hydroxyvitamin D levels | rs113140528 | T | A | 0.032 | 0.003 | 8.3e-35 |
| Serum 25-Hydroxyvitamin D levels | rs1660818 | A | G | 0.016 | 0.002 | 3.65e-14 |
| Serum 25-Hydroxyvitamin D levels | rs2276360 | C | G | 0.111 | 0.002 | 1e-200 |
| Serum 25-Hydroxyvitamin D levels | rs11182428 | C | T | -0.013 | 0.002 | 1.22e-10 |
| Serum 25-Hydroxyvitamin D levels | rs2171427 | A | G | -0.015 | 0.003 | 3.1e-08 |
| Serum 25-Hydroxyvitamin D levels | rs73413596 | C | T | 0.023 | 0.004 | 1.08e-09 |
| Serum 25-Hydroxyvitamin D levels | rs1038165 | T | C | 0.012 | 0.002 | 1.84e-09 |
| Serum 25-Hydroxyvitamin D levels | rs10859995 | C | T | -0.040 | 0.002 | 8.9e-91 |
| Serum 25-Hydroxyvitamin D levels | rs12317268 | G | A | -0.021 | 0.003 | 1.91e-14 |
| Serum 25-Hydroxyvitamin D levels | rs34284484 | G | T | -0.012 | 0.002 | 4.75e-08 |
| Serum 25-Hydroxyvitamin D levels | rs142004400 | C | A | -0.032 | 0.005 | 1.83e-09 |
| Serum 25-Hydroxyvitamin D levels | rs8018720 | C | G | -0.038 | 0.003 | 2.11e-49 |
| Serum 25-Hydroxyvitamin D levels | rs2756119 | A | G | 0.013 | 0.002 | 4.25e-10 |
| Serum 25-Hydroxyvitamin D levels | rs62012775 | T | A | -0.016 | 0.003 | 5.79e-10 |
| Serum 25-Hydroxyvitamin D levels | rs1800588 | T | C | -0.033 | 0.002 | 1.67e-43 |
| Serum 25-Hydroxyvitamin D levels | rs62007299 | A | G | -0.012 | 0.002 | 4.2e-08 |
| Serum 25-Hydroxyvitamin D levels | rs2123930 | A | G | -0.014 | 0.002 | 9.14e-11 |
| Serum 25-Hydroxyvitamin D levels | rs1532085 | G | A | 0.026 | 0.002 | 1.25e-38 |
| Serum 25-Hydroxyvitamin D levels | rs77924615 | A | G | -0.015 | 0.003 | 1.28e-09 |
| Serum 25-Hydroxyvitamin D levels | rs11076175 | G | A | 0.024 | 0.003 | 5.1e-20 |
| Serum 25-Hydroxyvitamin D levels | rs8063565 | C | G | 0.013 | 0.002 | 3.65e-09 |
| Serum 25-Hydroxyvitamin D levels | rs11542462 | A | G | -0.023 | 0.003 | 8.7e-16 |
| Serum 25-Hydroxyvitamin D levels | rs10083762 | G | C | 0.012 | 0.002 | 2.25e-08 |
| Serum 25-Hydroxyvitamin D levels | rs12949853 | A | G | 0.014 | 0.003 | 2.92e-08 |
| Serum 25-Hydroxyvitamin D levels | rs2952289 | T | C | 0.018 | 0.002 | 7.49e-13 |
| Serum 25-Hydroxyvitamin D levels | rs10454087 | T | C | -0.012 | 0.002 | 1.9e-08 |
| Serum 25-Hydroxyvitamin D levels | rs2659007 | A | G | 0.011 | 0.002 | 2.8e-08 |
| Serum 25-Hydroxyvitamin D levels | rs4121823 | A | T | -0.019 | 0.003 | 1.2e-11 |
| Serum 25-Hydroxyvitamin D levels | rs2037511 | A | G | 0.017 | 0.003 | 4.85e-11 |
| Serum 25-Hydroxyvitamin D levels | rs8091117 | A | C | -0.026 | 0.004 | 1.09e-10 |
| Serum 25-Hydroxyvitamin D levels | rs80204526 | A | C | -0.053 | 0.010 | 3.91e-08 |
| Serum 25-Hydroxyvitamin D levels | rs62115743 | T | C | 0.027 | 0.004 | 1.24e-13 |
| Serum 25-Hydroxyvitamin D levels | rs212100 | C | T | -0.066 | 0.003 | 1.38e-135 |
| Serum 25-Hydroxyvitamin D levels | rs142158911 | A | G | 0.026 | 0.003 | 2.55e-17 |
| Serum 25-Hydroxyvitamin D levels | rs429358 | C | T | -0.022 | 0.003 | 5.57e-16 |
| Serum 25-Hydroxyvitamin D levels | rs3745669 | C | T | -0.013 | 0.002 | 3.19e-10 |
| Serum 25-Hydroxyvitamin D levels | rs3814995 | T | C | -0.013 | 0.002 | 3.06e-09 |
| Serum 25-Hydroxyvitamin D levels | rs11606 | G | C | 0.011 | 0.002 | 1.65e-08 |
| Serum 25-Hydroxyvitamin D levels | rs8107974 | T | A | 0.039 | 0.004 | 1.84e-25 |
| Serum 25-Hydroxyvitamin D levels | rs8121940 | G | C | -0.038 | 0.002 | 1.88e-52 |
| Serum 25-Hydroxyvitamin D levels | rs8123293 | G | A | 0.028 | 0.003 | 5.19e-20 |
| Serum 25-Hydroxyvitamin D levels | rs2207132 | A | G | -0.037 | 0.005 | 1.34e-11 |
| Serum 25-Hydroxyvitamin D levels | rs2229742 | C | G | -0.025 | 0.003 | 1.12e-14 |
| Serum 25-Hydroxyvitamin D levels | rs5771043 | A | G | -0.012 | 0.002 | 3.46e-09 |
| Serum 25-Hydroxyvitamin D levels | rs6003465 | C | T | -0.012 | 0.002 | 7.12e-09 |
| Serum 25-Hydroxyvitamin D levels | rs2074735 | C | G | 0.027 | 0.004 | 7.89e-12 |

**Supplementary Table** **2** Heterogeneity, pleiotropy, and others.

| **Exposure** | **Outcome** | **R^2^** | **F** | **Heterogeneity** | **Pleiotropy** |
| --- | --- | --- | --- | --- | --- |
| Serum 25-Hydroxyvitamin D levels | Laryngeal cancer | 4.40% | 184 | 0.946 | 0.429 |

Exposure: Refers to the exposure variable in the analysis. In this case, it is “Serum 25-Hydroxyvitamin D levels”, which indicates that the researcher is analyzing the relationship between serum vitamin D levels and laryngeal cancer.

Outcome: Refers to the outcome variable in the analysis. In this case, it is “Laryngeal cancer”, which is the disease or health outcome of interest in the study.

R^2^: Indicates the proportion of variance in the outcome variable explained by the exposure variable. In this case, an R^2^ of 4.40% means that serum vitamin D levels explain 4.40% of the risk of laryngeal cancer.

F: This is the F-statistic, commonly used to detect the strength of the association between the exposure and outcome variables. An F value of 184 indicates a strong association, and generally, the larger the F value, the more significant the association.

Heterogeneity: Typically used to test whether there are significant differences among different genetic instrumental variables (such as SNPs). A heterogeneity value of 0.946 suggests that the effect differences among the SNPs are small and the heterogeneity is not significant.

Pleiotropy: The pleiotropy metric is used to assess whether genetic instrumental variables affect the outcome variable through pathways other than the exposure variable. A pleiotropy value of 0.429 suggests that in this case, pleiotropy is not significant, meaning that the genetic instrumental variables primarily influence laryngeal cancer risk through vitamin D levels.

**Supplementary Table 3** Heterogeneity assessment results of different methods.

| **Method** | **Q** | **Q_df** | **Q_pval** |
| --- | --- | --- | --- |
| MR Egger | 76.91 | 98 | 0.9432 |
| IVW | 77.54 | 99 | 0.9456 |

Notes:

Q Statistic (Q): Used to assess heterogeneity among SNPs. The higher the Q value, the greater the heterogeneity.

Degrees of Freedom (Q_df): The degrees of freedom for the Q statistic, usually equal to the number of SNPs minus 1.

Q Statistic p-value (Q_pval): Used to determine the significance of the Q statistic. If the p-value is greater than 0.05, it indicates no significant heterogeneity.

Summary: The p-values for the Q statistic in both methods are greater than 0.05, indicating no significant heterogeneity. This suggests that the analysis results from both methods are robust, and each SNP's contribution to the outcome is consistent.

**Supplementary Table 4** Directionality assessment results from egger regression.

| **Item** | **Value** |
| --- | --- |
| Egger Regression Intercept | 8.1e-06 |
| Standard Error | 1e-05 |
| Directionality p-value | 0.429 |

Notes:

Egger Regression Intercept: The intercept from Egger regression is used to detect directional pleiotropy. Directional pleiotropy occurs when genetic variants influence not only the exposure but also the outcome directly. If the intercept significantly deviates from zero (p-value < 0.05), it suggests the presence of directional pleiotropy.

Standard Error: The uncertainty of the intercept estimate. The smaller the standard error, the higher the precision of the estimate.

Directionality p-value: Used to assess whether the Egger regression intercept significantly deviates from zero. A p-value greater than 0.05 indicates no significant directional pleiotropy.

Summary:

The Egger regression intercept is close to zero, the standard error is small, and the directionality p-value is greater than 0.05, indicating no significant directional pleiotropy.

This result enhances the reliability of the causal inference, as directional pleiotropy can cause bias, but no significant directional pleiotropy was found in this analysis.

**Supplementary Table 5** Instrumental variables.

| **Exposure** | **SNP** | **effect_allele** | **other_allele** | **beta** | **SE** | **p-val** |
| --- | --- | --- | --- | --- | --- | --- |
| 25 hydroxyvitamin D level | rs72665698 | G | T | 0.014 | 0.002 | 1.99e-09 |
| 25 hydroxyvitamin D level | rs12123821 | T | C | 0.074 | 0.005 | 2.25e-59 |
| 25 hydroxyvitamin D level | rs867772 | G | A | -0.014 | 0.002 | 3.64e-11 |
| 25 hydroxyvitamin D level | rs61747728 | T | C | 0.032 | 0.005 | 4.41e-10 |
| 25 hydroxyvitamin D level | rs2934744 | C | A | 0.022 | 0.002 | 3.96e-26 |
| 25 hydroxyvitamin D level | rs61816766 | C | T | 0.084 | 0.006 | 1.09e-50 |
| 25 hydroxyvitamin D level | rs10908469 | C | A | 0.016 | 0.002 | 2.2e-13 |
| 25 hydroxyvitamin D level | rs7528419 | G | A | 0.019 | 0.002 | 2.41e-16 |
| 25 hydroxyvitamin D level | rs6698680 | G | A | -0.012 | 0.002 | 8.99e-10 |
| 25 hydroxyvitamin D level | rs3750296 | C | G | -0.021 | 0.002 | 2.09e-24 |
| 25 hydroxyvitamin D level | rs7519574 | A | G | 0.017 | 0.003 | 2.09e-11 |
| 25 hydroxyvitamin D level | rs10127775 | T | A | 0.012 | 0.002 | 3.43e-09 |
| 25 hydroxyvitamin D level | rs11127048 | A | G | 0.018 | 0.002 | 6.41e-19 |
| 25 hydroxyvitamin D level | rs7569755 | A | G | 0.014 | 0.002 | 8.03e-11 |
| 25 hydroxyvitamin D level | rs1800440 | C | T | -0.014 | 0.002 | 2.06e-08 |
| 25 hydroxyvitamin D level | rs2012736 | A | C | -0.046 | 0.004 | 7.65e-37 |
| 25 hydroxyvitamin D level | rs55886116 | T | G | 0.014 | 0.002 | 5.13e-09 |
| 25 hydroxyvitamin D level | rs1047891 | A | C | -0.014 | 0.002 | 1.16e-11 |
| 25 hydroxyvitamin D level | rs4635554 | G | T | -0.013 | 0.002 | 6.44e-10 |
| 25 hydroxyvitamin D level | rs6724965 | G | A | -0.017 | 0.003 | 1.29e-10 |
| 25 hydroxyvitamin D level | rs34293138 | C | T | -0.012 | 0.002 | 3.82e-09 |
| 25 hydroxyvitamin D level | rs6773343 | T | C | 0.013 | 0.002 | 5.2e-09 |
| 25 hydroxyvitamin D level | rs6438900 | G | C | 0.014 | 0.002 | 9.59e-10 |
| 25 hydroxyvitamin D level | rs1972994 | T | A | -0.018 | 0.002 | 7.99e-18 |
| 25 hydroxyvitamin D level | rs4364259 | A | G | 0.016 | 0.002 | 6.91e-11 |
| 25 hydroxyvitamin D level | rs4645189 | T | C | 0.023 | 0.003 | 2.79e-19 |
| 25 hydroxyvitamin D level | rs4694423 | A | C | -0.097 | 0.002 | 1e-200 |
| 25 hydroxyvitamin D level | rs58073039 | G | A | -0.014 | 0.002 | 2.16e-11 |
| 25 hydroxyvitamin D level | rs71601787 | A | G | 0.042 | 0.002 | 3.34e-92 |
| 25 hydroxyvitamin D level | rs7699711 | T | G | -0.029 | 0.002 | 6.97e-49 |
| 25 hydroxyvitamin D level | rs4616820 | T | C | -0.011 | 0.002 | 1.4e-08 |
| 25 hydroxyvitamin D level | rs116472025 | A | G | 0.037 | 0.006 | 6.83e-10 |
| 25 hydroxyvitamin D level | rs1229984 | C | T | -0.047 | 0.006 | 4.85e-13 |
| 25 hydroxyvitamin D level | rs78649910 | A | T | -0.018 | 0.003 | 4.32e-09 |
| 25 hydroxyvitamin D level | rs7657132 | G | A | -0.015 | 0.002 | 8.36e-13 |
| 25 hydroxyvitamin D level | rs7724488 | G | A | 0.012 | 0.002 | 4.11e-09 |
| 25 hydroxyvitamin D level | rs27774 | A | G | -0.012 | 0.002 | 1.64e-08 |
| 25 hydroxyvitamin D level | rs7718395 | G | C | 0.013 | 0.002 | 1.67e-09 |
| 25 hydroxyvitamin D level | rs466360 | A | G | -0.011 | 0.002 | 1.13e-08 |
| 25 hydroxyvitamin D level | rs12196316 | C | T | -0.012 | 0.002 | 5.51e-09 |
| 25 hydroxyvitamin D level | rs9476310 | T | C | 0.011 | 0.002 | 1.1e-08 |
| 25 hydroxyvitamin D level | rs2245133 | C | T | -0.021 | 0.003 | 1.62e-15 |
| 25 hydroxyvitamin D level | rs942380 | G | A | 0.011 | 0.002 | 7.84e-09 |
| 25 hydroxyvitamin D level | rs75741381 | G | C | -0.016 | 0.003 | 2.19e-09 |
| 25 hydroxyvitamin D level | rs1011468 | A | G | -0.014 | 0.002 | 1.35e-12 |
| 25 hydroxyvitamin D level | rs34452119 | A | G | -0.011 | 0.002 | 2.65e-08 |
| 25 hydroxyvitamin D level | rs10085881 | C | T | -0.014 | 0.002 | 8.02e-11 |
| 25 hydroxyvitamin D level | rs34726834 | T | C | 0.014 | 0.002 | 6.65e-10 |
| 25 hydroxyvitamin D level | rs7828742 | G | A | -0.022 | 0.002 | 3.06e-28 |
| 25 hydroxyvitamin D level | rs804280 | A | C | 0.013 | 0.002 | 4.43e-11 |
| 25 hydroxyvitamin D level | rs13284054 | C | T | 0.017 | 0.003 | 8.39e-09 |
| 25 hydroxyvitamin D level | rs10818769 | G | C | -0.017 | 0.003 | 3.35e-09 |
| 25 hydroxyvitamin D level | rs532436 | A | G | -0.015 | 0.003 | 2.17e-09 |
| 25 hydroxyvitamin D level | rs10887718 | T | C | -0.012 | 0.002 | 1.44e-10 |
| 25 hydroxyvitamin D level | rs2607863 | C | T | -0.025 | 0.004 | 1.02e-08 |
| 25 hydroxyvitamin D level | rs9423639 | T | C | -0.012 | 0.002 | 4.01e-08 |
| 25 hydroxyvitamin D level | rs146128209 | G | A | -0.047 | 0.004 | 1.03e-35 |
| 25 hydroxyvitamin D level | rs12803256 | G | A | 0.100 | 0.002 | 1e-200 |
| 25 hydroxyvitamin D level | rs12798050 | T | C | 0.107 | 0.003 | 1e-200 |
| 25 hydroxyvitamin D level | rs182244780 | A | G | -0.335 | 0.009 | 1e-200 |
| 25 hydroxyvitamin D level | rs61887417 | A | G | -0.034 | 0.006 | 1.42e-09 |
| 25 hydroxyvitamin D level | rs7930750 | T | C | -0.060 | 0.002 | 1.1e-194 |
| 25 hydroxyvitamin D level | rs11500197 | A | G | -0.012 | 0.002 | 3.99e-08 |
| 25 hydroxyvitamin D level | rs12417758 | C | T | 0.012 | 0.002 | 2.08e-09 |
| 25 hydroxyvitamin D level | rs138072379 | T | C | 0.043 | 0.007 | 6.56e-10 |
| 25 hydroxyvitamin D level | rs1792214 | G | T | 0.016 | 0.002 | 2.09e-15 |
| 25 hydroxyvitamin D level | rs964184 | C | G | 0.040 | 0.003 | 5.11e-44 |
| 25 hydroxyvitamin D level | rs2847500 | A | G | -0.021 | 0.003 | 7.79e-13 |
| 25 hydroxyvitamin D level | rs12317268 | G | A | -0.019 | 0.003 | 9.15e-12 |
| 25 hydroxyvitamin D level | rs10771090 | G | A | -0.011 | 0.002 | 4.32e-08 |
| 25 hydroxyvitamin D level | rs9668081 | T | C | 0.012 | 0.002 | 5.38e-09 |
| 25 hydroxyvitamin D level | rs10859995 | C | T | -0.039 | 0.002 | 7.03e-89 |
| 25 hydroxyvitamin D level | rs11830764 | C | G | 0.022 | 0.004 | 1.96e-08 |
| 25 hydroxyvitamin D level | rs9569235 | C | A | -0.013 | 0.002 | 6.1e-09 |
| 25 hydroxyvitamin D level | rs8018720 | C | G | -0.032 | 0.003 | 4.04e-36 |
| 25 hydroxyvitamin D level | rs7148857 | G | C | -0.013 | 0.002 | 3.93e-10 |
| 25 hydroxyvitamin D level | rs4267257 | G | A | -0.014 | 0.003 | 2.59e-08 |
| 25 hydroxyvitamin D level | rs7178572 | G | A | -0.014 | 0.002 | 1.62e-11 |
| 25 hydroxyvitamin D level | rs17651741 | A | G | -0.013 | 0.002 | 4.9e-08 |
| 25 hydroxyvitamin D level | rs174418 | C | T | 0.022 | 0.002 | 2.99e-28 |
| 25 hydroxyvitamin D level | rs1800588 | T | C | -0.030 | 0.002 | 2.65e-36 |
| 25 hydroxyvitamin D level | rs8063706 | T | A | 0.013 | 0.002 | 3.64e-09 |
| 25 hydroxyvitamin D level | rs77924615 | A | G | -0.016 | 0.002 | 1.46e-10 |
| 25 hydroxyvitamin D level | rs3816117 | C | T | -0.016 | 0.002 | 2.63e-17 |
| 25 hydroxyvitamin D level | rs11542462 | A | G | -0.022 | 0.003 | 3.39e-14 |
| 25 hydroxyvitamin D level | rs35733741 | A | G | 0.012 | 0.002 | 3.81e-09 |
| 25 hydroxyvitamin D level | rs2909218 | T | C | 0.017 | 0.002 | 2.81e-12 |
| 25 hydroxyvitamin D level | rs4121823 | A | T | -0.016 | 0.003 | 1.3e-09 |
| 25 hydroxyvitamin D level | rs2037511 | A | G | 0.016 | 0.003 | 9.29e-10 |
| 25 hydroxyvitamin D level | rs8091117 | A | C | -0.024 | 0.004 | 1.03e-09 |
| 25 hydroxyvitamin D level | rs3814995 | T | C | -0.015 | 0.002 | 2.83e-12 |
| 25 hydroxyvitamin D level | rs73015021 | G | A | 0.023 | 0.003 | 1.15e-14 |
| 25 hydroxyvitamin D level | rs1048328 | A | G | 0.028 | 0.004 | 4.12e-15 |
| 25 hydroxyvitamin D level | rs424132 | A | T | -0.012 | 0.002 | 8.74e-09 |
| 25 hydroxyvitamin D level | rs58542926 | T | C | 0.032 | 0.004 | 8.57e-19 |
| 25 hydroxyvitamin D level | rs112285002 | T | C | 0.060 | 0.003 | 1.77e-110 |
| 25 hydroxyvitamin D level | rs5112 | G | C | -0.015 | 0.002 | 2.12e-12 |
| 25 hydroxyvitamin D level | rs6127099 | T | A | -0.037 | 0.002 | 9.3e-62 |
| 25 hydroxyvitamin D level | rs2762938 | A | G | 0.013 | 0.002 | 4.63e-11 |
| 25 hydroxyvitamin D level | rs2229742 | C | G | -0.026 | 0.003 | 7.13e-16 |
| 25 hydroxyvitamin D level | rs960596 | T | C | 0.012 | 0.002 | 2.23e-09 |
| 25 hydroxyvitamin D level | rs2074735 | C | G | 0.027 | 0.004 | 6.55e-12 |

**Supplementary Table 6** Heterogeneity, pleiotropy, and others.

| **Exposure** | **Outcome** | **R^2^** | **F** | **Heterogeneity** | **Pleiotropy** |
| --- | --- | --- | --- | --- | --- |
| 25 hydroxyvitamin D level | Laryngeal cancer | NA | 143 | 0.594 | 0.873 |

**Supplementary Table 7** Heterogeneity assessment results of different methods.

| **Method** | **Q** | **Q_df** | **p-val** |
| --- | --- | --- | --- |
| MR Egger | 89.12 | 92 | 0.5657 |
| Inverse variance weighted | 89.14 | 93 | 0.5939 |

**Supplementary Table 8** Directionality assessment results from egger regression.

| **Egger regression intercept:** | -1.9e-06 |
| --- | --- |
| **Standard error:** | 1.2e-05 |
| **Directionality p-value:** | 0.873 |

**Supplementary Table 9** F-statistics.

| **Conditional** | **F-statistics** |
| --- | --- |
| ebi-a-GCST90000616 | 78.13141 |
| ieu-b-4877 | 20.25829 |

**Supplementary Table 10**

[EXCEL]

**Supplementary Table 11**

[EXCEL]

**Supplementary Table 12**

[EXCEL]

**Supplementary Table 13**

[EXCEL]

**Supplementary Figure 1**


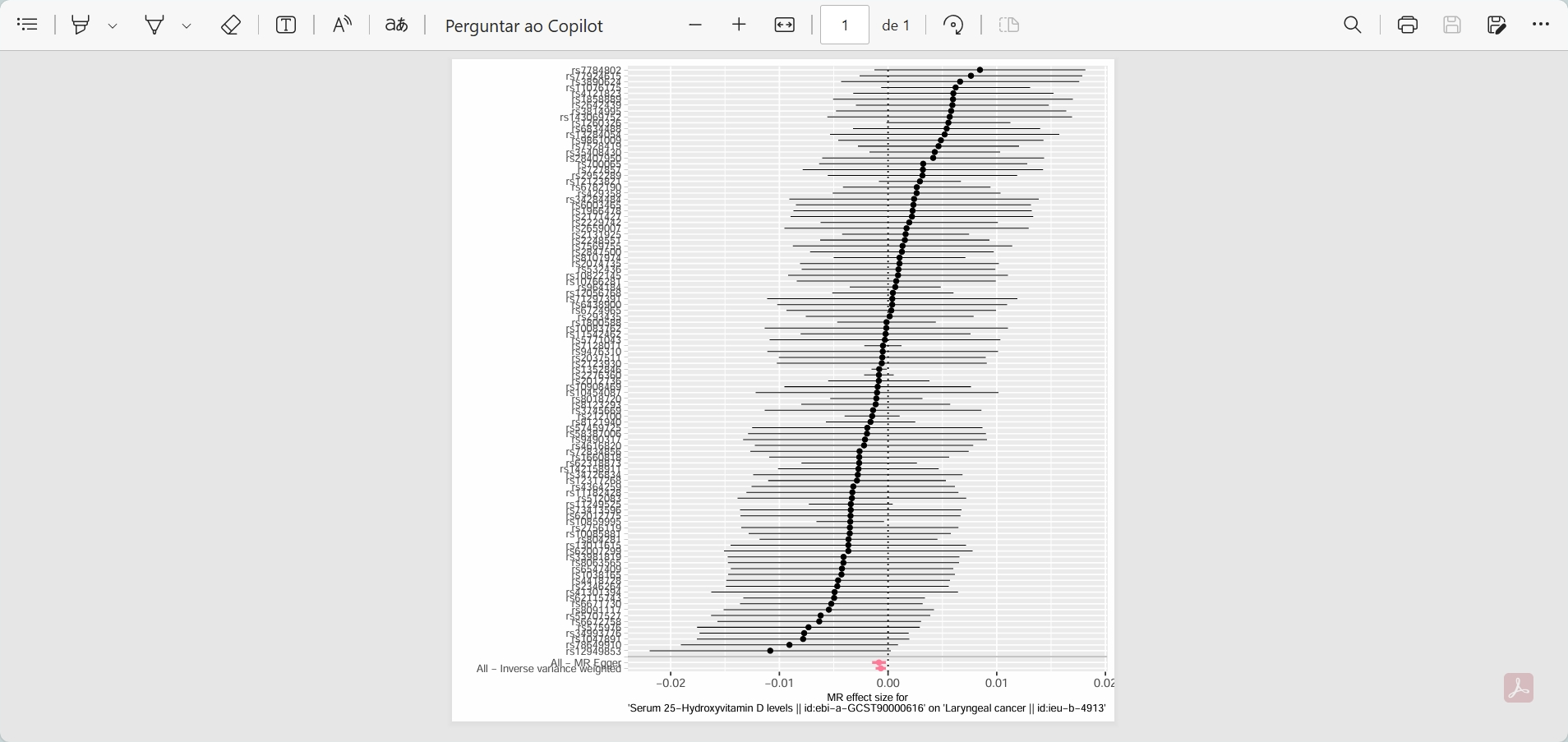


**Supplementary Figure 2**


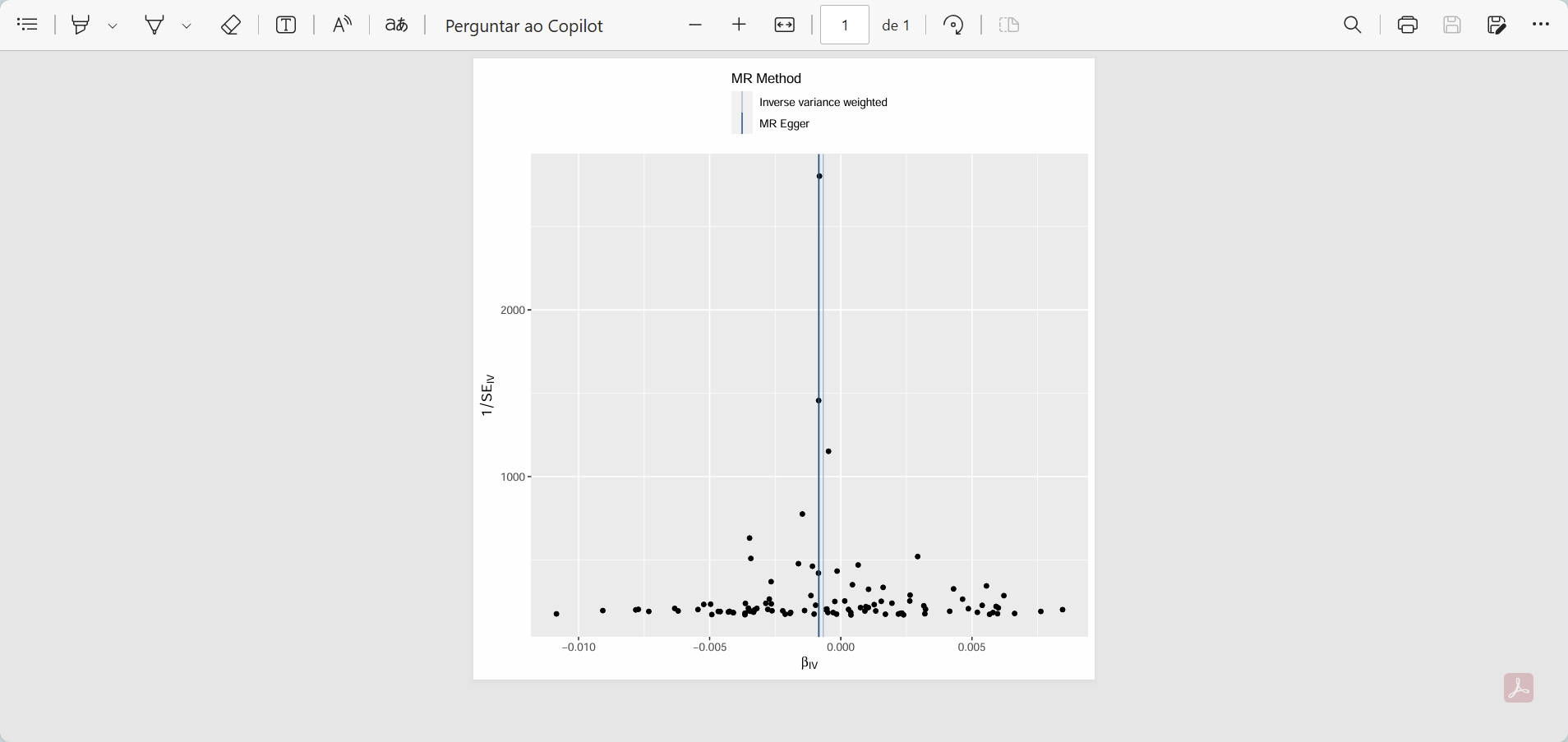


**Supplementary Figure 3**


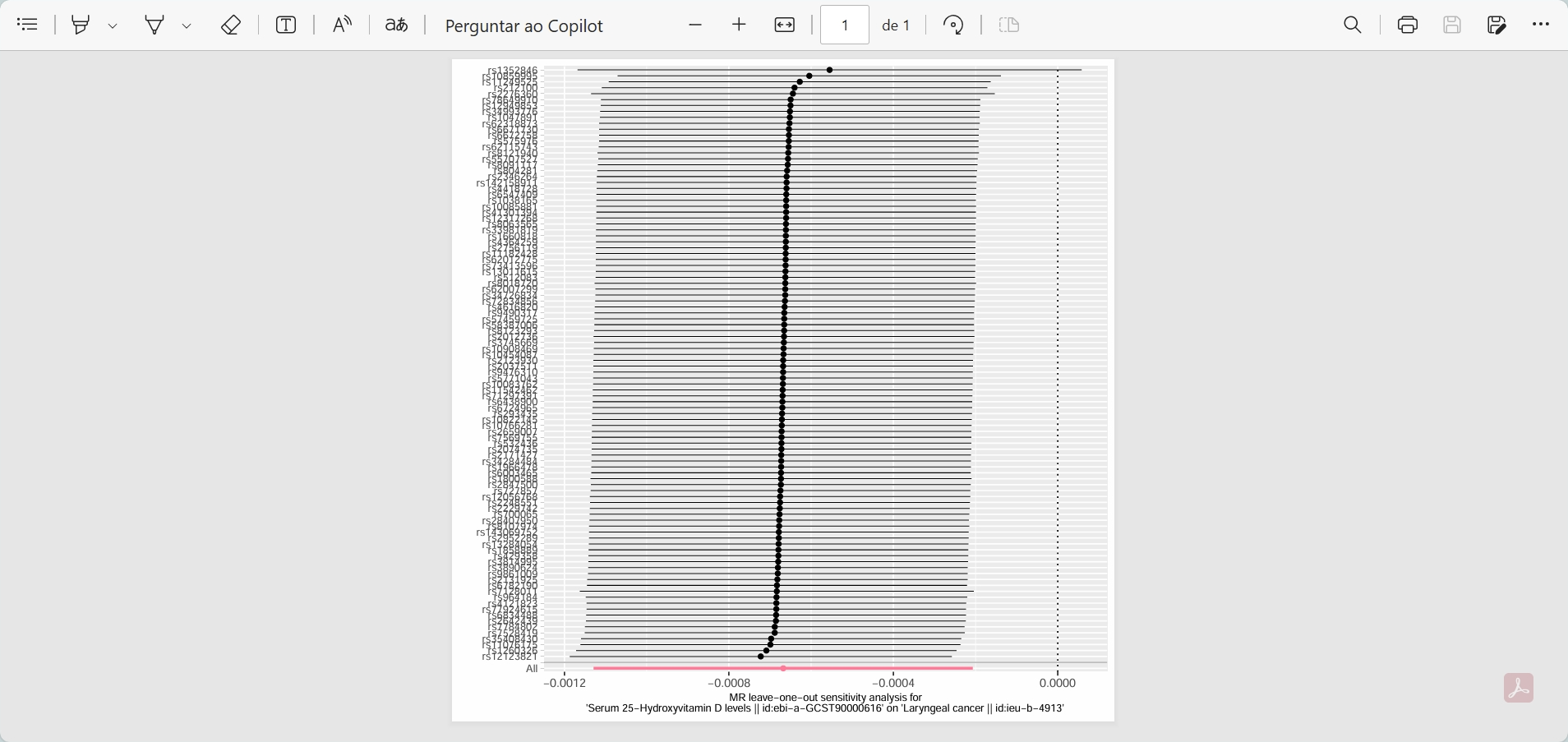


**Supplementary Figure 4**


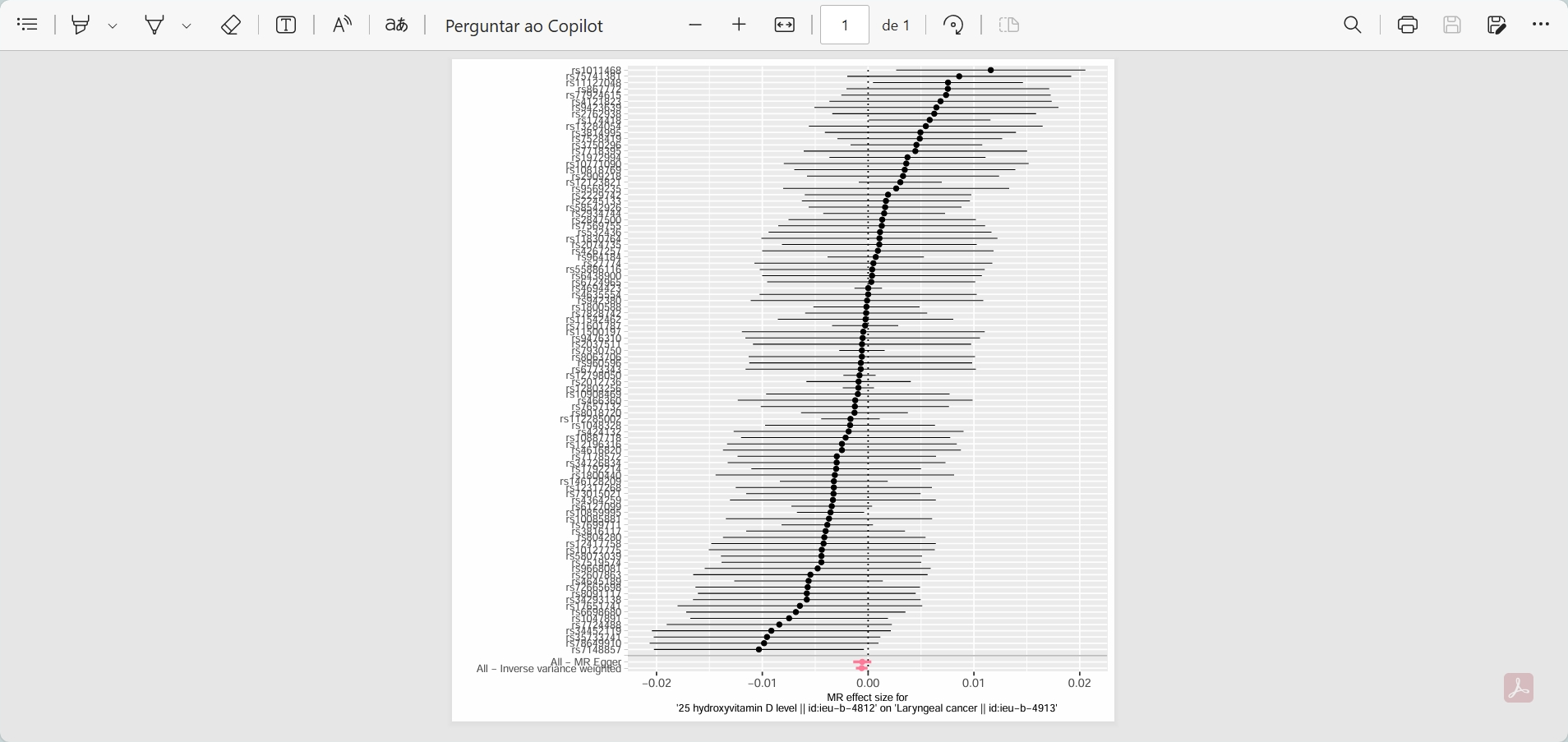


**Supplementary Figure 5**


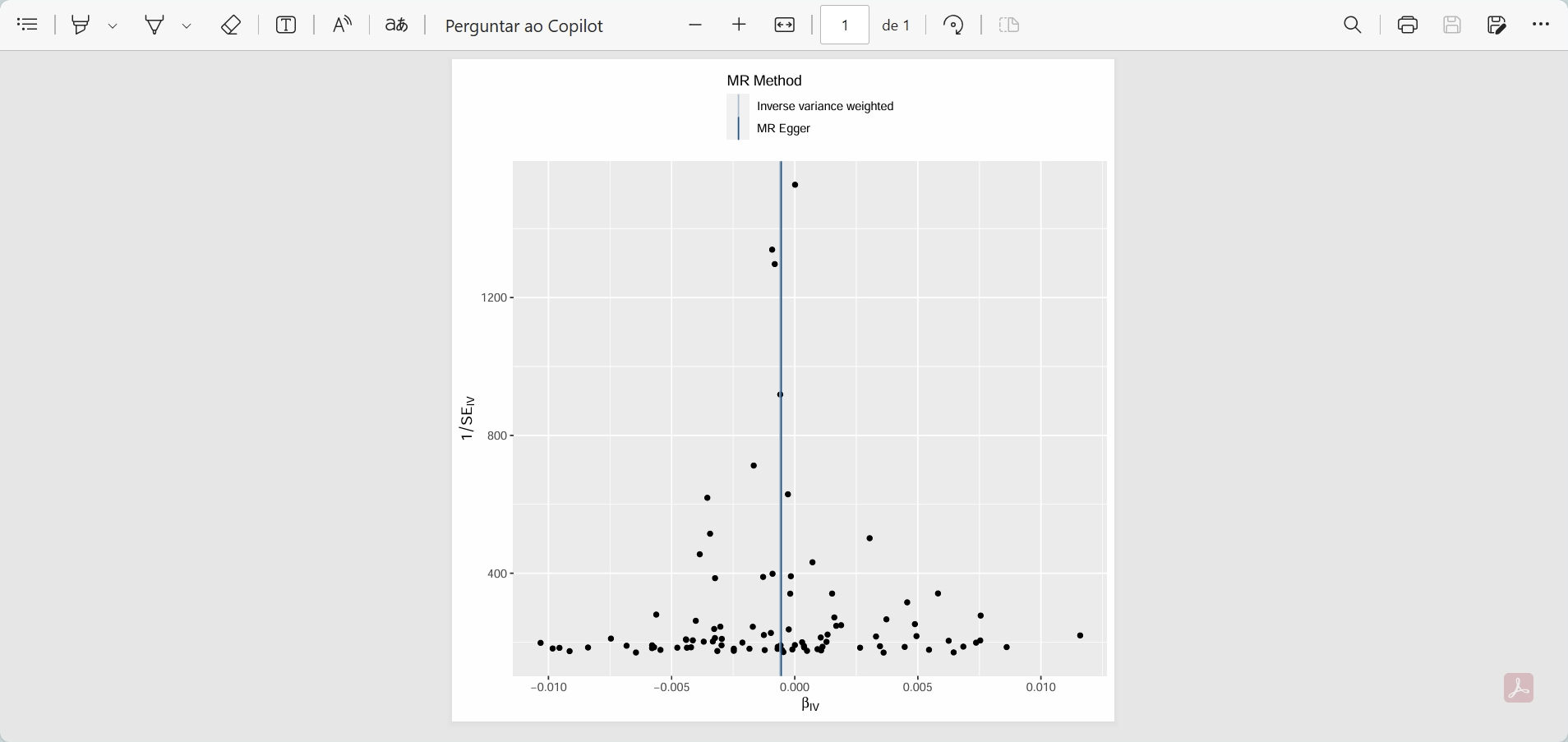


**Supplementary Figure 6**


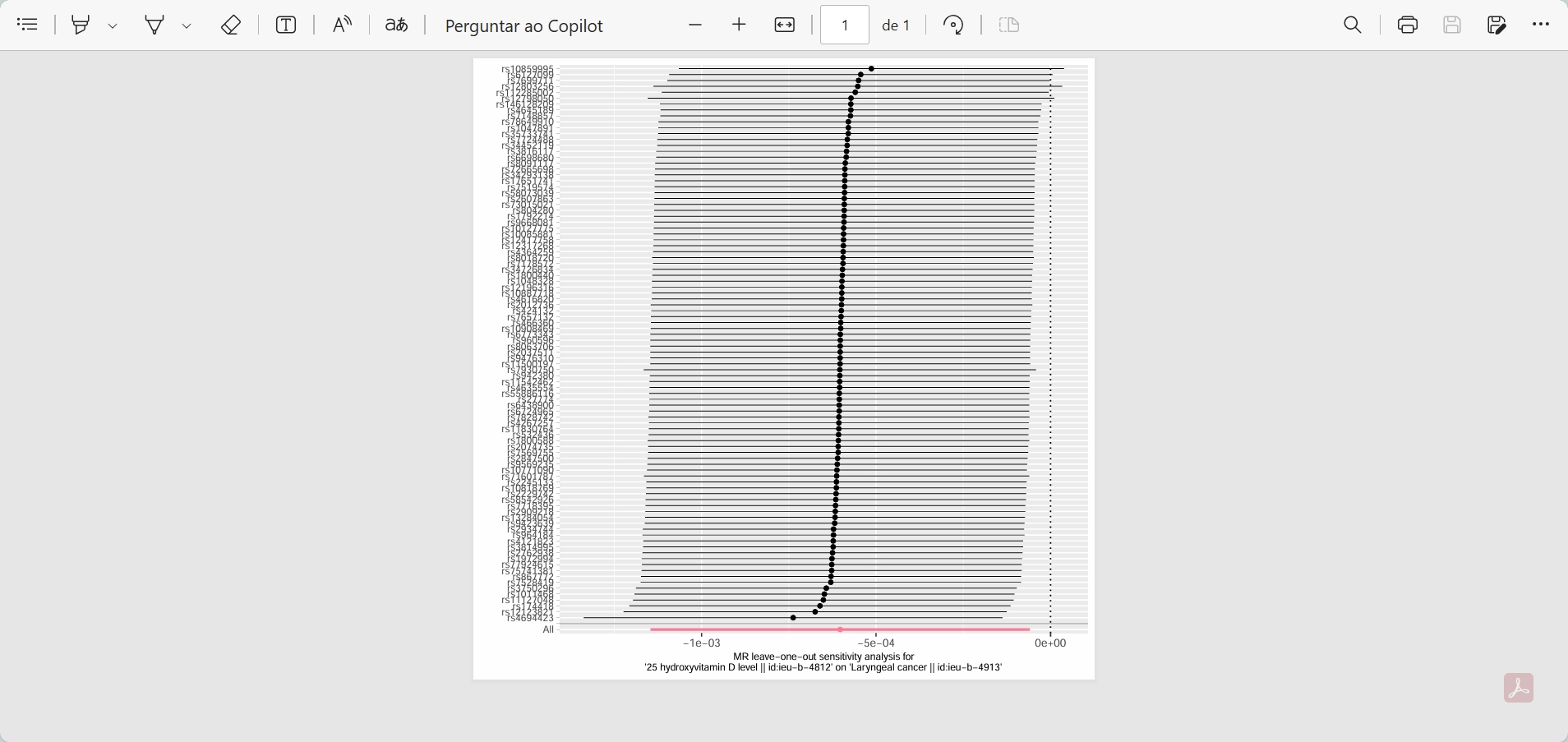

Supplement: Supplementary file 1 [file mmc1.docx]
